# Reformulation and Priorities for Reducing Energy Density; Results from a Cross-Sectional Survey on Fat Content in Pre-Packed Cakes and Biscuits Sold in British Supermarkets

**DOI:** 10.3390/nu11061216

**Published:** 2019-05-28

**Authors:** Roberta Alessandrini, Feng J. He, Kawther M. Hashem, Monique Tan, Graham A. MacGregor

**Affiliations:** Wolfson Institute of Preventive Medicine, Barts and The London School of Medicine & Dentistry, Queen Mary University of London, Charterhouse Square, London EC1M 6BQ, UK; f.he@qmul.ac.uk (F.J.H.); k.hashem@qmul.ac.uk (K.M.H.); m.tan@qmul.ac.uk (M.T.); g.macgregor@qmul.ac.uk (G.A.M.)

**Keywords:** saturated fat, total fat, energy density, calories, sugar, reformulation, cakes, biscuits

## Abstract

Cakes and biscuits contribute to energy, total and saturated fat and sugar in British diets. So far, the UK government has prompted manufacturers to reduce energy density in these products through a reduction of their sugar content. We conducted a cross-sectional survey of the fat content of cakes and biscuits available in nine UK supermarket chains. In cakes (*n* = 381), the mean total fat content was 17.9 ± 5.2 g/100 g (39% of the overall energy); range (1.4–35.6 g/100 g) and the average saturated fat content in cakes was 5.9 ± 3.4 g/100 g (13% of the overall energy); range (0.3–20 g/100 g). In biscuits (*n* = 481), the mean total fat content was 21.8 g ± 6.3 g/100 g (40% of the overall energy); range (0.7–38.9 g/100 g) and the average saturated fat content was 11.4 ± 4.9 g/100 g (23% of the overall energy); range (0.3–22.3 g/100 g). In both cakes and biscuits, total and saturated fat content was positively correlated with energy density. Our results show that cakes and biscuits sold in UK supermarkets are high in total and saturated fat, and that fat content contributes substantially to product energy density. Fat reformulation in these products would effectively reduce energy density, calorie intake and help prevent obesity. Fat reformulation should be implemented simultaneously with sugar reformulation and be focused on saturated fat, as this will have the additional effect of lowering LDL cholesterol.

## 1. Introduction

Obesity and overweight prevalence are high in England. The most recent statistics show that two out of three adults and one out of three children are overweight or obese [1]. Although individuals have some responsibility in making healthier food choices, the food environment plays a determinant role in influencing these choices [2,3]. Many countries are now making efforts towards improving the nutritional quality of the food supply chain. To align with these principles, the UK government has also committed to engage with the food industry to reduce calories in food and drinks through product reformulation [4].

The concept of reformulation is not new, as in the UK and in many other countries, reformulation has already been used to successfully reduce the amount of salt and industrially-produced trans-fatty acids in widely consumed processed foods [5,6]. Since 2015, Public Health England, the agency appointed by the UK government to lead on the first ever sugar reformulation program, has focused mainly on lowering calories through sugar reduction (the Sugar Reduction Program) [7]. However, a recent evaluation of the Sugar Reduction Program has shown that since its implementation, product energy density (i.e., kcal/100 g) has been minimally reduced [8]. The reason may be that when manufacturers reduce sugar content, the starch and protein content is increased proportionally. Starch and protein have the same energy density as sugar (~4 kcal/g), and thus a reduction in sugar content may not automatically translate into a fall in energy density.

Fat (including saturated fat) are the most energy-dense nutrients, providing on average 9 kcal/g [9], and fat reformulation (i.e., reduction of product fat content) could result in a considerable reduction in energy density. To achieve a reduction in energy density, the fat and sugar content of products should be replaced by lower calorie ingredients, such as dietary fiber. 

Recently, Public Health England has been working beyond the concept of calorie reduction through sugar reformulation and has laid out the Calorie Reduction Program [10]. The strategy challenges the food industry to achieve a 20% reduction of the calories in products which contribute significantly to the population calorie intake by 2024. Reformulation has been suggested as one of the main approaches for the industry to adopt. However, the plan does not include yet any guideline on how to effectively reduce food energy density. Moreover, sweet categories such as cakes, biscuits and chocolate confectionery are not included in the Calorie Reduction Program because they are included in the Sugar Reduction Program [8].

In the past, cakes and biscuits were considered occasional treats and consumed infrequently; however, this is now not the case, with nine out of ten people reporting regular consumption [11,12]. Cakes and biscuits (including pastries, buns and fruit pies) cumulatively contribute to 9–15% of the total energy intake of the British population [13]. These products also contribute to 8–12% of the total fat and 14–23% of the total dietary free sugars [13].

Cakes and biscuits are also important sources of saturated fat, contributing to 9–15% of the total saturated fat intake in the population [13]. In England, saturated fat intake is high, and exceeds the recommended limit of 10% of the food energy. The most recent UK National Diet and Nutrition Survey reported that average saturated fat intake in children, adolescents and adults was around 12–13% of the total food energy; in older adults, the energy contribution from saturated fat was 14.6% of the total food energy [13]. These figures, which are based on self-reported data, are most likely to be underestimated as under-reporting is very common in these types of surveys [14,15,16].

In our previous paper, we reported on the sugar and energy content of cakes and biscuits sold in British supermarkets [17]. In this paper, we aim to evaluate the amount of fat and saturated fat in the same sample of products; moreover, we will evaluate the contribution of fat and sugar to product energy density. The results will provide evidence on the most effective way of reducing energy density in cakes and biscuits.

## 2. Materials and Methods

A cross-sectional survey of biscuits and cakes was carried out in 2016. At that time, the primary aim of the survey was to evaluate the sugar content and energy density of biscuits and cakes. The data on total and saturated fat for the same products were also collected. The full survey methodology has been described in detail and published elsewhere [17]. Some of the methods relevant to the present report are briefly described here.

Total and saturated fat data were taken from the product nutrition information panel. We collected data from products sold in nine stores. Each store belonged to one of the following supermarket chains: Aldi, Asda, Lidl, Morrisons, Sainsbury’s, Tesco, The Co-operative, Waitrose and Marks & Spencer. These nine chains jointly hold more than 90% of the grocery market share in the UK [18]. We assumed our sample can be considered representative of pre-packed cakes and biscuits available on the British market. The data were collected on one occasion for each supermarket in the London metropolitan area. To obtain the largest possible sample, large stores were chosen instead of smaller stores.

### 2.1. Inclusion and Exclusion Criteria

We included own-label (i.e., those products produced for a supermarket chain) and branded (i.e., those made by a well-known manufacturer, and which have the manufacturer’s label on them) cakes and biscuits. We also included low-fat cakes and biscuits (i.e., products with a low-fat claim). We excluded savory biscuits, crackers and crispbreads, which are often grouped with cakes and biscuits. We also excluded in-store self-service bakery items as they typically lack nutrition information at the point of purchase, and any product without a nutrition information panel.

### 2.2. Product Categorization

All the cakes and biscuits products had been previously categorized, and the related data has been published elsewhere [17]. In short, products were categorized according to their product description and their formulation (e.g., shortbread biscuit or Battenberg cake) [17]. Not all the products fit in specific categories; some were left uncategorized and excluded from the within-category analysis. Data for all the products (categorized and uncategorized) were included in the general analysis and contributed to the overall results. The products were also categorized as supermarket own-label or branded products.

### 2.3. Analysis

We reported descriptive statistics (mean, SD, range) for total fat, saturated fat (g/100 g) and energy density (kcal/100 g) for all the cakes and all the biscuits included and for each category. Subsequently, we calculated the mean percentage of energy contribution from total and saturated fat and sugar for cakes and biscuits. To assess whether there was a significant difference between the fat content of the own-label and branded products, we performed independent samples t-tests.

We compared the total fat and the saturated fat content of each product with the criteria used for the color coding adopted in the UK Front of Pack Labelling [19]. For total fat, products containing ≤3 g/100 g are classified as green/low, products with >3 g and ≤17.5 g/100 g are classified as amber/medium, and products ≥17.5 g/100 g as red/high [20]. For saturated fat, products containing ≤1.5 g/100 g are classified as green/low, products with >1.5 g and ≤5 g /100 g are classified as amber/medium, and products ≥5 g/100 g as red/high. We calculated the ratio of all cakes and biscuits that would get a green/amber/red code for total and saturated fat [20]. We performed a Pearson’s correlation analysis between total fat, saturated fat, sugars (g/100 g) and energy density (kcal/100 g). All the analyses were performed using IBM SPSS Statistics 25.

## 3. Results

### 3.1. Nutrient Content and Contribution to Product Energy Density

On average, the cakes (*n* = 381) had an energy density of 406 ± 37 kcal/100 g and an average total fat content of 17.9 ± 5.2 g/100 g, which contributed to 39% of the overall product energy. The average sugar content was 36.6 g/100 g, which contributed to 34% of the overall product energy. The average saturated fat content was 5.9 ± 3.4 g/100 g, which contributed to 13% of the overall product energy (Table 1). On average, the biscuits (*n* = 481) had an energy density of 484 ± 38 kcal/100. The average total fat content was 21.8 ± 6.3 g/100 g, which contributed to 40% of the overall product energy. The average content of sugar was 30.0 ± 9.2 g/100 g, which contributed to 23% of the overall product energy. The average saturated fat content was 11.4 ± 4.9 g/100 g, which contributed to 23% of the overall product energy (Table 1).

### 3.2. Comparison with the UK Front of Pack Labelling Guidelines

**Figure 1:**
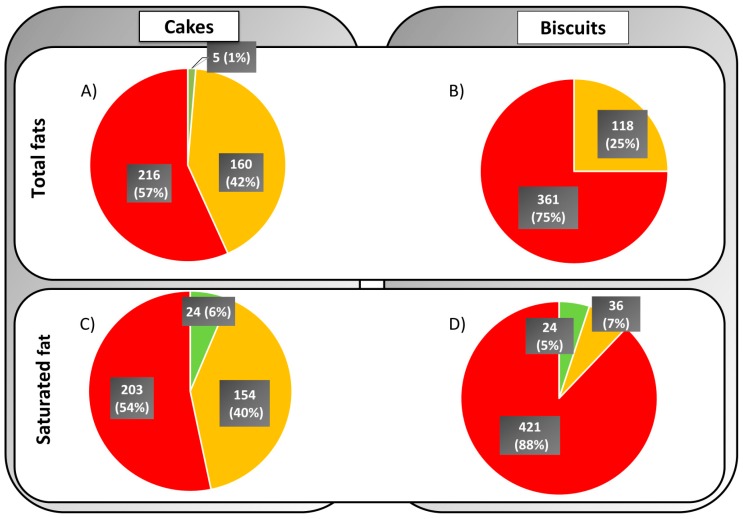
Percentage of cakes (**A**,**C**) and biscuits (**B**,**D**) that would receive a low/medium/high criteria for total fat (**A**,**B**) and saturated fat (**C**,**D**).

When looking at data for cakes, fifty-seven percent would receive a red (high) color code for total fat, while only 1% would receive a green (low) color code (Figure 1A). Similarly, fifty-four percent would receive a red (high) color code for saturated fat, while only 6% would receive a green (low) color code (Figure 1C). Seventy-five percent of the biscuit products would receive a red (high) color code for total fat (Figure 1B), while only 0.41% (2 products, not shown in Figure 1B) would receive a green (low) color code. Eighty-eight percent of biscuit products would receive a red (high) color code for saturated fat, while only 5% would receive a green (low) color code (Figure 1D).

### 3.3. Variation in Total and Saturated Fat and Energy Density

#### 3.3.1. Cakes

**Figure 2:**
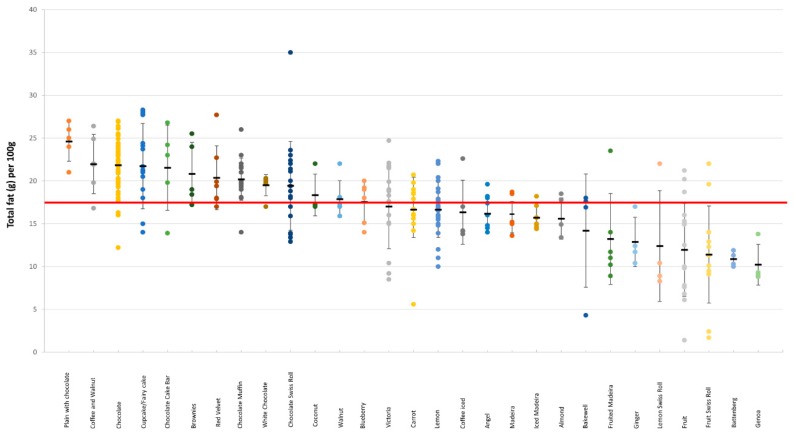
Total fat content (g/100 g) in different categories of cakes. The red line denotes the high criteria for total fat.

**Figure 3:**
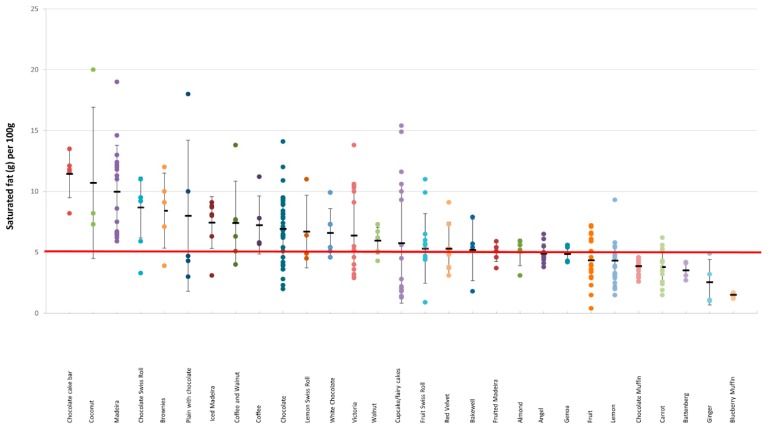
Saturated fat content (g/100 g) in different categories of cakes. The red line denotes the high criteria for saturated fat.

There was a considerable variation in the total and saturated fat content within each cake category (Figure 2 and Figure 3). For example, in Bakewell products total fat content ranged from 4.3 to 18 g/100 g, and in Carrot cakes total fat content ranged from 5.6 to 29.7 g; in Fruit Swiss Rolls, the amount of saturated fat per 100 g ranged from 0.9 to 11 g, and in Lemon cakes from 1.5 g to 9.9 g (see Appendix A, Table A1). The categories with the highest energy density were also the same categories with the highest total fat content. There was no significant difference between branded and own-label products (18 g vs. 17.8 g, *p* = 0.72 and 6.3 g vs. 5.7 g, *p* = 0.16 for total and saturated fat, respectively) (Appendix A, Table A1).

#### 3.3.2. Biscuits

**Figure 4:**
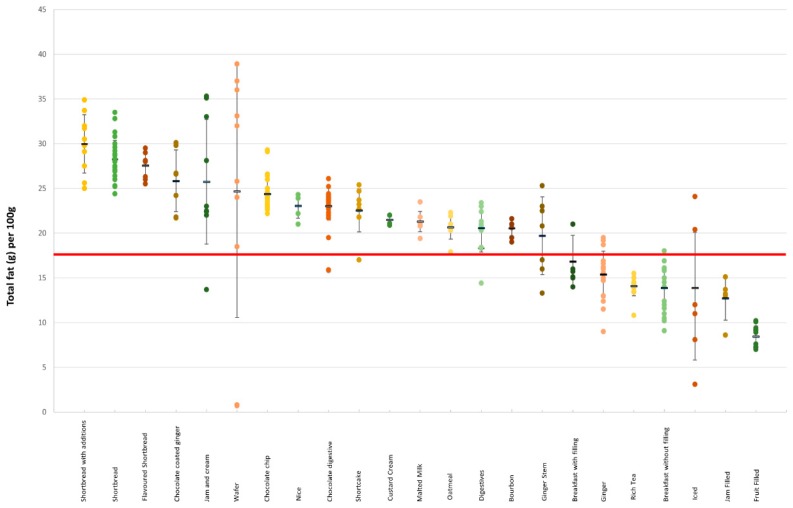
Total fat content (g/100 g) in different categories of biscuits. The red line denotes the high criteria for total fat.

**Figure 5:**
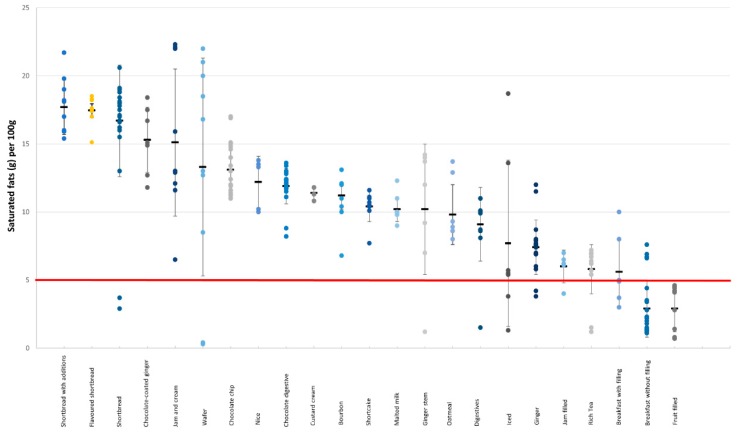
Saturated fat content (g/100 g) in different categories of biscuits. The red line denotes the high criteria for saturated fat.

There was a large variation in the total fat and saturated fat content among all the products but also within each category (Figure 4 and Figure 5). For example, in rich tea biscuits, the amount of saturated fat per 100 g spanned from 1.2 to 7.2 g (see Appendix A, Table A2). Own-label products had, on average, higher amounts of total fat and saturated fat compared to branded products (22.8 g vs. 20.3 g, *p* > 0.001 and 12.3 g vs. 10 g, *p* > 0.001, respectively) (Appendix A, Table A2).

### 3.4. Correlations

**Figure 6:**
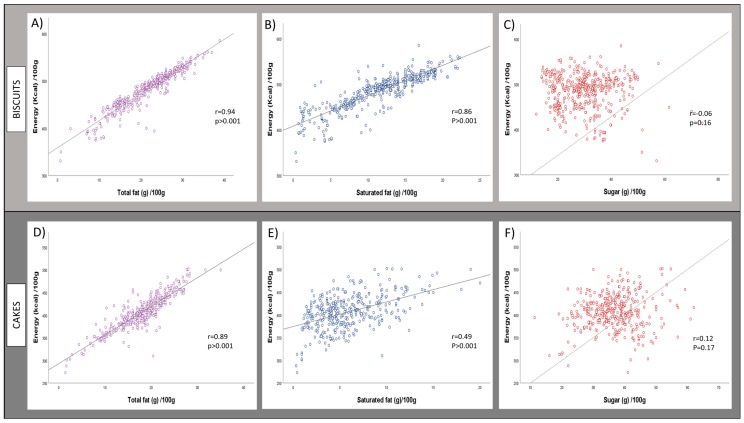
Correlations between total fat, saturated fat and sugar (g/100) and energy density (kcal/100 g) in biscuits (**A**–**C**) and cakes (**D**–**F**).

In both biscuits and cakes, there was a significant positive correlation between total fat content and energy density (*r* = 0.94, *p* < 0.001 for biscuits and *r* = 0.89, *p* < 0.001 for cakes) (Figure 6A,D). There was also a significant correlation between saturated fat content and energy density (*r* = 0.86, *p* < 0.001 for biscuits and *r* = 0.49, *p* < 0.001 for cakes) (Figure 6B,E). The correlations between sugar content and energy density were weak and not significant (*r* = −0.06, *p* = 0.16 for biscuits and *r* = 0.12 and *p* = 0.17 for cakes) (Figure 6C,F).

## 4. Discussion

Cakes and biscuits are foods that are widely consumed by children and adults. In 2016/2017 the UK average per capita consumption of cakes and biscuits (including buns, pastries and crispbreads) was 319 g per week [20]. According to our analysis, only a very small proportion (less than 5%) of the surveyed products would receive a green (low) label for total and saturated fat [20]. As previously reported, these products are also very high in sugar, with 97% of the cakes surveyed and 74% of the biscuits surveyed receiving a red (high) label for sugar [17].

The correlation analysis revealed that in both cakes and biscuits, total and saturated fat, but not sugar, was strongly correlated with energy density, i.e., the higher the content of total and saturated fat, the higher the energy density. Our analysis has also shown that, in both cakes and biscuits, total and saturated fat content contributed to about 20% of the product weight and around 40% of the overall energy. On the other hand, sugar contributed to more than 30% of the product weight for both cakes and biscuits, and 23% of biscuits’ overall energy and 34% of cakes’ overall energy. These findings show that fat reformulation can be more effective in lowering energy density than sugar reformulation alone, although both should be implemented.

The category analysis showed that the categories highest in fat were also the highest in energy density. Moreover, we observed a large variation in total and saturated fat content within the same product category. This finding clearly indicates that reformulation to reduce total and saturated fat and energy density is possible as some manufacturers are already producing products with a more healthful nutrient composition and lower energy density.

### 4.1. Strengths and Weaknesses of the Study

Our study included a large number of products (almost 400 cakes and 500 biscuits), classified in well-defined categories. Categorizing products was a cumbersome task, but helped to observe the large variation in fat, sugar and energy density, which demonstrates that reformulation within each category is entirely feasible. Products in different categories, such as chocolate cakes or fruit cakes, have very different recipes and we believe that it is important to show the extent to which reformulation is possible within every category.

The present survey gathered data provided on product packaging and we thus relied on their precision and accuracy. Although the manufacturers are responsible for reporting accurate, up-to-date and precise information about product nutrients and energy density, there is a lack of publicly available reports or studies investigating the accuracy of such information in the UK. The results of such investigations would be a valuable resource for validating the results of product surveys such as the present one, and most of all, for providing accurate information to the consumers.

This study did not include products from the out-of-home sector (e.g., cakes from artisanal shops or in-store bakery items) because the provision of nutrition information is not mandatory in these sectors [21]. It could be assumed that our results are conservative since the requirement to display nutrition information may directly influence manufacturers to produce products with fewer calories and fat.

### 4.2. Comparison with Other Studies

Our findings are similar to those reported by the UK Department of Health in 2008, which reported data on the total and saturated fat content of 402 products sold in British outlets. Their sample included not only cakes and biscuits but also other categories such as pastries, flapjacks, scones, doughnuts and savory biscuits [22]. Although the Department of Health data comprised a smaller sample size, it is possible to observe that total and saturated fat content in biscuits and cakes has not changed in the last ten years [22]. Two small studies from Portugal and India reported lower total and saturated fat content in biscuits compared to those observed in our study. However, the representativeness of their samples is questionable [23,24]. One Malaysian study reported similar levels of total and saturated fat in biscuits compared to the present findings [25]. It was not possible to find total and saturated fat data for cakes. This is because cakes are a less well-defined food category than biscuits and are often grouped with other bakery products.

### 4.3. Implications

Since 2003, reformulation has successfully been implemented in the UK to reduce salt in a variety of widely consumed processed foods [5]. Many countries have followed the lead of the UK salt reduction model and implemented their own national salt reduction programs. One of the key aspects of the salt reduction strategy is that reformulation has been implemented gradually and unobtrusively so that consumers do not notice the salt reduction in their everyday foods, such as bread. The other key aspect was the setting of reformulation targets to be achieved in a defined timeframe.

The Sugar Reduction Program also adopted reformulation as one of its key strategies to reduce sugar and energy density in manufactured food and drink products [8]. However, the reformulation efforts have failed to reduce energy density in cakes and biscuits [8], possibly because their sugar content has not been replaced with non-calorific bulk replacers such as dietary fiber. A recent modeling study has shown that if the Sugar Reduction Program were entirely implemented across the UK, there would be a reduction of energy intake of 25 kcal per person per day [26]. Such reduction is minimal compared to the 200–300 excess calories consumed daily by the average British person [10]. Our results indicate that fat reformulation is an essential mechanism to meaningfully reduce product energy density and achieve a substantial deficit of energy intake. 

Although salt and sugar/fat reformulation share the same underlying principle (i.e., the reduction of unhealthy nutrients and components), fat and sugar reformulation are more complex than salt reformulation. This is because salt reformulation consists of the removal of milligrams of salt from foods, while fat and sugar contribute to the weight of the product. The fat and sugar of solid foods should be substituted with minimally calorific ingredients, such as fruit and vegetable residue, which is high in dietary fiber.

Fat and sugar reformulation should not be viewed as two opposing approaches to reduce food energy density and calorie intake in the population. Reducing product sweetness is an important public health strategy to prevent tooth decay and weight gain [27]. Evidence shows that energy delivered as sugar is inadequately compensated (i.e., the intake of other foods and drinks is not voluntarily and effectively reduced), which causes excessive energy intake, and therefore, weight gain over time [27]. So far, the Sugar Program has failed to deliver substantial results for solid foods but it has led to some progress in sugary drinks. This may be due to multiple reasons The first is that when sugar is reduced in drinks, the volume of the product (and therefore the product portion size) does not change; second, non-sugar sweeteners can be easily used for conferring sweetness to drinks; and third, the UK Soft Drinks Industry Levy (SDIL) has been effective in pressuring manufacturers to reformulate [8].

In view of the success of the SDIL in reducing the sugar content of sugary drinks, we recommend the implementation of an energy density tax to effectively reduce product energy density in solid food [28]. At the moment, the Calorie Reduction Program only recommends the food industry to reduce calories on a voluntary basis. Setting clear guidelines and targets on how to reduce not only sugar and calories but also fat could result in more substantial progress, even without a regulatory framework. Moreover, the setting of targets would provide a level playing field for the industry, as has been done in the Salt Reduction Program.

Saturated fat intake in the British population is high, and if fat reformulation was focused on saturated fat reduction, this would have the additional benefit of improving population health via an independent effect of LDL cholesterol reduction [29,30]. Some saturated-rich fat such as butter (51% saturated fat) and palm oil (49% saturated fat), which are widely used in cakes and biscuits manufacturing, have a greater environmental impact than most vegetable oils [31,32]. Their substitution in food products could therefore benefit population health (via a reduction in LDL cholesterol) but also improve the overall environmental sustainability of the food supply chain.

The findings of this study could potentially apply not only to cakes and biscuits but also to other solid “sweet” categories high in fat and sugar (e.g., morning goods, puddings and chocolate confectionery). Total and saturated fat reformulation (by substitution with fruit and vegetable residue) could be the most effective strategy to reduce energy density also in other foods such as sausages, bread with additions, pizza and ready meals.

## 5. Conclusions

Our study demonstrates that biscuits and cakes sold in UK supermarkets contain a considerable amount of fat and saturated fat, and that fat contributes substantially to product energy density. Our survey also showed a large variation in the total and saturated fat content within each cake and biscuit category; the categories highest in total fat were often the highest in energy density. Additionally, in both cakes and biscuits, total and saturated fat content was positively correlated with energy density, but sugar content was not. Taken together, these findings suggest that reducing fat could result in a substantial reduction of product energy density. In addition, a reduction in food energy density from saturated fat could result in an overall reduction of obesity and LDL cholesterol in the British population. Public Health England is in the process of developing the Calorie Reduction Program, in which cakes and biscuits (despite being widely consumed foods) have not been included, only because these categories are already included in the Sugar Program. Sugar reformulation alone can be an effective strategy for energy density reduction in sugary drinks, but it should not be the sole instrument used to reduce energy density in solid foods such as cakes and biscuits. Our work demonstrates that to reduce food energy density effectively, it is important to simultaneously reduce both sugar and fat (particularly saturated fat). The findings of this study could be extended to other sweet solid food categories.

## Figures and Tables

**Table 1:** Energy density, total and saturated fat and sugar content (mean, SD), and their respective percentage energy contribution to overall product energy in cakes and biscuits.

	N	Energy Density * (kcal/100 g)	Fat (g/100)	% Energy from Fat	Sugar * (g/100g)	% Energy from Sugar
Cakes	381	406 ± 37	17.9 ± 5.2 of which saturated 5.9 ± 3.4	39 % from saturated 13	36.6 ± 7.6	34
Biscuits	481	484 ± 38	21.8 ± 6.3 of which saturated 11.4 ± 4.9	40 % from saturated 23	30.0 ± 9.2	23

* Energy density and sugar figures from Hashem et al. [17].

## Appendix A

**Table A1:** Total and saturated fat content and energy density in different categories of cakes, mean ± SD (range).

Category	N	Total Fat (g/100 g)	Saturated Fat (g/100 g)	Energy Density * (kcal/100 g)
Own-label	290	17.8 ± 4.9 (1.4–35.0)	5.7 ± 3.2 (0.4–20.0)	404 ± 35 (273–502)
Branded	91	18.0 ± 6.2 (1.6–31.7)	6.3 ± 3.9 (0.3–18.0)	414 ± 42 (288–500)
Plain with chocolate	5	24.6 ± 2.3 (21.0–27.0)	8.0 ± 6.2 (3.0–18.0)	446 ± 16 (421–457)
Coffee and Walnut	6	22.0 ± 3.5 (16.8–26.4)	7.4 ± 3.4 (4.0–13.8)	433 ± 22 (403–460)
Chocolate	42	21.8 ± 3.1 (12.2–27.5)	6.9 ± 2.6 (2.0–14.1)	430 ± 21 (365–475)
Cupcakes/Fairy Cakes	19	21.7 ± 5.2 (14.6–28.3)	5.8 ± 4.9 (1.3–15.4)	440 ± 41 (380–502)
Chocolate Cake Bar	5	21.5 ± 5.4 (13.9–26.8)	11.4 ± 2.0 (8.2–13.5)	445 ± 41 (376–484)
Brownies	5	20.8 ± 3.7 (17.2–25.5)	8.4 ± 3.1 (3.9–12.0)	430 ± 23 (406–454)
Red Velvet	7	20.3 ± 3.7 (17.7–27.7)	5.3 ± 2.2 (3.1–9.1)	433 ± 27 (411–489)
Chocolate Muffins	18	20.2 ± 2.5 (14.0–26.0)	3.9 ± 0.6 (2.6–4.6)	416 ± 21 (369–475)
White Chocolate	6	19.5 ± 1.2 (17.0–20.3)	6.6 ± 2.0 (4.6–9.9)	423 ± 12 (402–436)
Chocolate Swiss Rolls	18	19.4 ± 5.2 (12.9–35.3)	10.0 ± 3.6 (5.9–19.0)	420 ± 36 (366–500)
Coconut	4	18.3 ± 2.4 (17.5–22.0)	10.7 ± 6.2 (7.3–20.0)	416 ± 36 (394–470)
Walnut	6	17.8 ± 2.1 (15.9–22.8)	6.0 ± 1.1 (4.3–7.3)	405 ± 11 (395–426)
Blueberry Muffins	6	17.5 ± 2.4 (14.5–20.0)	1.5 ± 0.2 (1.2–1.7)	378 ± 28 (331–408)
Victoria	18	17.0 ± 4.9 (8.5–24.7)	6.4 ± 3.4 (2.9–13.8)	402 ± 36 (346–456)
Carrot	16	16.8 ± 3.5 (5.6–29.7)	3.8 ± 1.4 (1.5–6.2)	389 ± 20 (323–415)
Lemon	21	16.6 ± 3.3 (16.7–22.3)	4.3 ± 2.1 (1.5–9.9)	394 ± 22 (358–439)
Coffee	5	16.3 ± 3.7 (13.8–22.6)	7.2 ± 2.4 (5.7–11.2)	403 ± 19 (391–435)
Angel	12	16.1 ± 2.6 (14.7–19.6)	4.9 ± 0.8 (3.8–6.5)	398 ± 16 (378–420)
Madeira	9	16.1 ± 2.2 (13.6–19.1)	8.7 ± 2.5 (3.3–11.0)	387 ± 9(367–395)
Iced Madeira	7	15.7 ± 1.4 (14.4–18.2)	7.4 ± 2.1 (3.1–9.1)	405 ± 20 (391–445)
Almond	5	15.6 ± 2.4 (13.4–18.5)	5.0 ± 1.1 (3.1–5.9)	396 ± 16 (379–411)
Bakewell	4	14.2 ± 6.6 (4.3–18.0)	5.2 ± 2.5 (1.8–7.9)	397 ± 41 (335–422)
Fruited Madeira	6	13.2 ± 5.3 (8.9–23.5)	5.0 ± 0.8 (3.7–5.9)	380 ± 51 (347–484)
Ginger	4	12.8 ± 2.9 (17.4–17.5)	2.6 ± 1.9 (1.0–4.9)	383 ± 20 (362–406)
Lemon Swiss Roll	4	12.4 ± 6.5 (8.3–22.0)	6.7 ± 3.0 (4.5–11.0)	375 ± 34 (349–425)
Fruit	17	11.9 ± 5.4 (1.4–21.2)	4.4 ± 2.0 (0.4–7.2)	367 ± 39 (273–449)
Fruit Swiss Roll	13	11.4 ± 5.7 (1.7–22.1)	5.3 ± 2.9 (0.9–11.0)	365 ± 36 (301–422)
Battenberg	4	10.8 ± 7.9 (15.0–11.9)	3.5 ± 0.7 (2.7–4.2)	401 ± 22 (375–421)
Genoa	4	10.2 ± 2.4 (8.8–13.8)	4.9 ± 0.7 (4.2–5.6)	356 ± 16 (344–380)
All products	381	17.9 ± 5.2 (1.4–35.6)	5.9 ± 3.4 (0.3–20.0)	406 ± 37 (273–502)

* Energy density figures from Hashem et al. [17].

**Table A2:** Total and saturated fat content and energy density indifferent categories of biscuits, mean ± SD (range).

Category	N	Total Fat (g/100)	Saturated Fat (g/100 g)	Energy Density * (kcal/100 g)
Own-label	296	22.8 ± 5.7 (5.7–35.3)	12.3 ± 4.6 (0.9–22.3)	490 ± 35(375–558)
Branded	185	20.3 ± 6.8 (6.8–38.9)	10.0 ± 5.2 (0.3–22)	474 ± 41(331–585)
Shortbread with additions	10	30.0 ± 3.3 (25.0–34.9)	17.7 ± 2.0 (15.4–21.7)	528 ± 18 (496–554)
Shortbread	28	28.3 ± 2.1 (24.4–33.5)	16.7 ± 4.1 (2.9–21.2)	519 ± 11 (497–553)
Flavored shortbread	8	27.6 ± 1.5 (25.5–29.5)	17.5 ± 1.1 (15.1–18.5)	519 ± 9 (595–532)
Chocolate-coated ginger	7	25.8 ± 3.4 (21.7–30.1)	15.3 ± 2.4 (11.8–18.4)	505 ± 23 (466–534)
Jam and cream	10	25.8 ± 7.0 (13.7–35.3)	15.1 ± 5.4 (6.5–22.3)	505 ± 39 (425–558)
Wafer	10	24.7 ± 14.1 (0.7–38.9)	13.3 ± 8.0 (0.3–22)	498 ± 89 (331–585)
Chocolate chip	29	24.4 ± 1.8 (22.2–29.3)	13.1 ± 2.0 (11–17)	498 ± 10 (485–522)
Nice	5	23.1 ± 1.4 (21.0–24.3)	12.2 ± 1.9 (10–13.8)	497 ± 7 (487–575)
Chocolate digestives	31	23.0 ± 2.2 (15.8–26.1)	11.9 ± 1.3 (8.2–13.6)	495 ± 13 (456–512)
Shortcake	9	22.5 ± 2.4 (17.0–25.4)	10.4 ± 1.1 (7.7–11.6)	490 ± 1 (458–532)
Custard cream	6	21.5 ± 0.6 (20.9–22)	11.4 ± 0.5 (10.8–11.8)	492 ± 3 (487–494)
Malted milk	9	21.3 ± 1.1 (19.4–23.5)	10.2 ± 0.9 (9–12.3)	489 ± 7 (476–597)
Oatmeal	8	20.7 ± 1.3 (17.9–22.3)	9.8 ± 2.2 (8–13.7)	478 ± 11 (454–491)
Digestives	11	20.6 ± 2.7 (14.4–23.4)	9.1 ± 2.7 (1.5–11)	481 ± 14 (447–498)
Bourbon	9	20.5 ± 1.0 (19.0–21.6)	10.2 ± 4.8 (1.2–14.2)	480 ± 9 (469–487)
Ginger stem	7	19.7 ± 4.3 (13.3–25.3)	10.2 ± 4.8 (1.2–14.2)	466 ± 28 (432–582)
Breakfast filled	7	16.8 ± 2.9 (14.0–21)	5.6 ± 2.5 (3–10)	455 ± 22 (433–497)
Ginger	19	15.4 ± 2.6 (9.0–19.5)	7.4 ± 2.0 (3.8–12)	456 ± 17 (421–489)
Rich Tea	16	14.1 ± 1.1 (10.8–15.5)	5.8 ± 1.8 (1.2–7.2)	454 ± 7 (436–467)
Breakfast unfilled	22	13.9 ± 2.4 (9.1–18)	2.9 ± 2.1 (1–7.6)	432 ± 19 (395–461)
Iced	7	13.0 ± 7.1 (3.1–24.1)	7.7 ± 6.1 (1.3–18.7)	451 ± 38 (399–515)
Jam filled	5	12.7 ± 2.4 (8.6–15.1)	6 ± 1.2 (4–7)	426 ± 18 (396–445)
Fruit filled	13	8.4 ± 1.2 (7.0–10.2)	2.9 ± 1.7 (0.7–4.6)	391 ± 1 (375–411)
All products	481	21.8 ± 6.3 (0.7–38.9)	11.4 ± 4.9 (0.3–22.3)	484 ± 38 (331–585)

* Energy density figures from Hashem et al. [17].
